# The Source of *Rag5*-Mediated Resistance to Soybean Aphids Is Located in the Stem

**DOI:** 10.3389/fpls.2021.689986

**Published:** 2021-07-16

**Authors:** Kumud Joshi, Joshua L. Baumgardner, Madison MacPhail, Shailesh R. Acharya, Elizabeth Blotevogel, Franck E. Dayan, Punya Nachappa, Vamsi J. Nalam

**Affiliations:** ^1^Department of Biological Sciences, Bowling Green State University, Bowling Green, OH, United States; ^2^Department of Agricultural Biology, Colorado State University, Fort Collins, CO, United States

**Keywords:** soybean, *Aphis glycines*, RAG5, stem resistance, antixenosis, antibiosis

## Abstract

The soybean aphid (*Aphis glycines*) continues to threaten soybean production in the United States. A suite of management strategies, such as planting aphid-resistant cultivars, has been successful in controlling soybean aphids. Several *Rag* genes (resistance against *A. glycines*) have been identified, and two are currently being deployed in commercial soybean cultivars. However, the mechanisms underlying *Rag*-mediated resistance are yet to be identified. In this study, we sought to determine the nature of resistance conferred by the *Rag5* gene using behavioral, molecular biology, physiological, and biochemical approaches. We confirmed previous findings that plants carrying the *Rag5* gene were resistant to soybean aphids in whole plant assays, and this resistance was absent in detached leaf assays. Analysis of aphid feeding behaviors using the electrical penetration graph technique on whole plants and detached leaves did not reveal differences between the *Rag5* plants and Williams 82, a susceptible cultivar. In reciprocal grafting experiments, aphid populations were lower in the *Rag5/rag5* (Scion/Root stock) chimera, suggesting that *Rag5-*mediated resistance is derived from the shoots. Further evidence for the role of stems comes from poor aphid performance in detached stem plus leaf assays. Gene expression analysis revealed that biosynthesis of the isoflavone kaempferol is upregulated in both leaves and stems in resistant *Rag5* plants. Moreover, supplementing with kaempferol restored resistance in detached stems of plants carrying *Rag5*. This study demonstrates for the first time that *Rag5*-mediated resistance against soybean aphids is likely derived from stems.

## Introduction

The soybean aphid (*Aphis glycines* Matsumura), an invasive pest, is a significant threat to soybean production in the United States (Hurley and Mitchell, 2017). Feeding injury due to soybean aphids results in stunted plant growth, leaf yellowing and wrinkling, reduced photosynthesis, and low pod fill and seed quality, resulting in low yields (Beckendorf et al., 2008). Soybean aphids also cause additional losses, as they are competent vectors of many economically important plant viruses, such as soybean mosaic virus and alfalfa mosaic virus (Hill et al., 2012). If left untreated, yield losses of up to 40% can occur because of severe infestations (Ragsdale et al., 2007; Rhainds et al., 2008). The economic impact of soybean aphids in North America has been estimated to $3.6–4.9 billion every year (Hill et al., 2012). A suite of integrated pest management strategies, such as prophylactic neonicotinoid seed treatment, development of economic thresholds and injury levels, and deployment of aphid-resistant cultivars, has been successful in controlling soybean aphids (Ragsdale et al., 2011; Krupke et al., 2017). However, continuous use of insecticides increases production costs and can lead to insecticide resistance (Hanson et al., 2017) and has adverse effects on non-target and beneficial insects (Desneux et al., 2007).

A cost-effective and sustainable strategy for managing aphids is host plant resistance (Ragsdale et al., 2011; Hodgson et al., 2012). Aphid-resistant varieties carrying resistance to *A. glycines* (*Rag*) genes have been available for commercial cultivation since 2010 (Hesler et al., 2013). Screening of soybean germplasm and plant introductions (PIs) for aphid resistance has led to the identification of 12 *Rag* genes (Hesler et al., 2013; Neupane et al., 2019; Natukunda and MacIntosh, 2020) and four quantitative trait loci (QTLs) (Bhusal et al., 2017). The *Rag* genes present antibiosis (adverse effect on insect biology or performance), antixenosis (non-host preference), and tolerance (similar yield in presence or absence of soybean aphids) as mechanisms of resistance. The best described *Rag* gene is *Rag1*, a dominant gene that provides antibiosis and antixenosis against soybean aphids (Hill et al., 2006a,b; Kim et al., 2010a). Although none of the *Rag* genes have been cloned, many have been mapped, and their chromosomal location is known. Fine mapping and high-resolution linkage analyses of the genomic regions containing *Rag* genes have identified nucleotide-binding site leucine-rich repeat (NLR) genes, the most numerous and common *R* genes in plants (Cui et al., 2015), as candidates for *Rag1* (Kim et al., 2010a) and *Rag2* (Kim et al., 2010b; Brechenmacher et al., 2015).

Besides the NLR genes, additional genes and mechanisms have also been proposed for other *Rag* genes (Lee et al., 2017). The *Rag5* gene identified in plant introduction (PI) 567301B located on chromosome 13 near the *Rag2* locus (Jun et al., 2012) is an example. Despite the proximity of the two genes, evidence suggests that the two genes segregate independently. The *Rag5*-containing QTL explains 50% of the phenotypic variance to aphid resistance (Jun et al., 2012). Aphid resistance observed in whole plants of *Rag5-*containing PI 567301B is lost on detached leaves (Michel et al., 2010), suggesting that resistance is induced in plant parts other than the leaves. A role for plant parts, such as roots, which are not under attack by herbivores, has been reported in several instances (Nalam et al., 2012, 2013; Fragoso et al., 2014; Agut et al., 2016). As for the nature of resistance conferred by *Rag5*, greenhouse and field experiments indicate antixenosis as compared with antibiosis observed in *Rag2*-containing plants (Mian et al., 2008; Jun et al., 2012). More recently, Lee et al. (2017) analyzed global changes in gene expression in response to aphid infestation in *Rag5* and/or *Rag5-*containing near-isogenic lines (NILs). In both NILs, aphid feeding resulted in activation of reactive oxygen species, upregulation of jasmonate signaling and the phenylpropanoid pathway, increased secondary cell wall synthesis, and down-regulation of photosynthesis.

Chemical defenses play a crucial role in plant response to insect herbivores, and several classes of secondary metabolites have been shown to impact aphid infestations adversely (Züst and Agrawal, 2016; Erb and Kliebenstein, 2020). In soybeans, phytoalexins, such as isoflavones, are induced in response to various stresses and serve as critical defensive compounds (Hart et al., 1983; Piubelli et al., 2003; Jahan et al., 2020). Isoflavones are a group of flavonoids found predominantly in legumes. A common theme in *Rag*-based soybean defenses is the upregulation of genes involved in flavonoid biosynthesis. In plants carrying the *Rag1* gene, aphid colonization induces isoflavone biosynthesis (Li et al., 2008) and accumulation in leaves (Hohenstein et al., 2019). Metabolic analysis of *Rag2* NILs indicates a correlation of aphid resistance with two triterpenoid saponins (isoflavones). In *Rag5* NILs, aphid resistance was correlated with three specific kaempferol glycosides (Mian, 2014). A triglucoside of kaempferol containing gentiobioside and sophorose linkages was 7-fold higher in resistant NILs than the susceptible NIL (Mian, 2014). A QTL associated with aphid resistance in soybeans is also correlated with a locus for high isoflavone content (Meng et al., 2011), providing additional evidence for the role of isoflavones in soybean response to aphids. Although isoflavones have antimicrobial properties, their role in defenses against aphids has not been extensively characterized and warrants further investigation.

There is little knowledge of the potential mechanisms underlying *Rag*-mediated resistance. A better understanding of the resistance mechanisms can provide information about candidate gene identities and help guide breeding efforts in the long term. In this study, we explore the nature of *Rag5*-mediated resistance to soybean aphids. We show that the *Rag5* gene confers both antibiosis and antixenosis modes of resistance against soybean aphids, and that the source of this resistance is likely located in the stem. Further, we show that the isoflavone, kaempferol, may be involved in reducing aphid populations in *Rag5* plants. Overall, this study provides the first evidence of stems as a potential source of *Rag5*-mediated resistance.

## Materials and Methods

### Seed Source and Plant Growth Conditions

Seeds for *Rag5*-containg PI 567301B and susceptible PI 548631(Williams 82) were obtained from the US National Plant Germplasm System, University of Illinois, Urbana, IL, United States. Plants were grown in Mastermix® 830 soilless media (Mastermix, Quakertown, PA, United States) in a growth chamber at 60–70% relative humidity, temperature of 24 ± 1°C, and photoperiod of 16:8 (L:D) hours (h) at photosynthetically active radiation (PAR) of 460 μmol/m^2^/s. The plants were watered three times per week and fertilized with Miracle Gro® (Scott's Co. LLC, Marysville, OH, United States) once a week. Soybean plants at the V1 stage [vegetative stage 1; full developed trifoliate leaf at the node above the unifoliate nodes based on the phenology scale described by Ritchie et al. (1985)] were used for all the experiments.

### Insect Colony

The lab colony of soybean aphids (biotype 1) was initially collected (~100–200 mixed-age individuals) from a soybean field at the Pinney Purdue Agricultural Center (PPAC), Watanah, Indiana. Biotype 1 aphids are avirulent and cannot overcome *Rag1*-conditioned resistance. The cultivar AG3432® (Bayer Crop Science, Kansas City, MO, United States), devoid of any seed treatment (naked seed), was used to maintain the insect colony. In the laboratory, the aphids were maintained on AG3432 at a temperature of 24 ± 1°C and a photoperiod of 16:8 (L:D) h in a 30 × 30 × 76 cm insect cage (BioQuip®, Rancho Dominguez, CA, United States). The colony was replenished with fresh plants (V1 to V4 stage) every 4–5 days. Apterous aphids were transferred to experimental plants with a fine-bristled paintbrush.

### Aphid Performance on Whole Plants, Detached Leaves, and Detached Stem + Leaves

#### Whole Plant Assays

A no-choice assay was performed on whole plants to determine aphid performance on *Rag5*-containing PI 567301B and the two susceptible controls, Williams 82 and AG3432. Ten adult (1-week-old) apterous aphids per trifoliate leaf were placed on all three leaflets in the first trifoliate leaf (30 adults/plant). A strip of Vaseline® was placed on the petiole of the trifoliate leaf to prevent the aphids from moving onto other parts of the plant (Unilever). The total number of adults and nymphs was counted every day for the following 4 days. We used 4 days because as per McCornack et al. (2004) and own observations, it takes, on average, 2 days for soybean aphid populations to double. The experiments were repeated three times (three independent experiments), with five biological replicates of each genotype per experiment.

#### Detached Leaf Assay

The detached leaf assay was conducted, as previously described by Michel et al. (2010). Briefly, a single trifoliate leaf was excised from the plant along with its petiole. Soybean plants at the V1 stage were used as the source of leaves. The petiole was carefully inserted in a 2-ml microfuge tube that contained 1.5 ml of water to maintain the moisture status of the leaf, and sealed with parafilm. The water in the microfuge tube was replenished as necessary to account for loss due to transpiration. Ten adult apterous aphids were placed on each detached leaf. The growth of the aphid populations was monitored for 4 days, during which the total number of aphids and the number of nymphs and adults were counted. Williams 82 and AG3432 served as the susceptible controls. The experiment was repeated three times over 3 months, with five replicates of each genotype in each experiment.

#### Detached Stem + Leaves Assay

A setup similar to the one used for the detached leaf assay was used for the detached stem + leaves assay. Soybean plants at the V1 stage served as the source for stems. The plants were excised ~2 cm below the base of the first trifoliate, allowing for a portion of the stem to be included and placed in a 50-ml centrifuge tube containing 25 ml of water and sealed with parafilm. A no-choice assay was performed by placing 10 adult apterous aphids on each leaf of the trifoliate and aphid populations, and the total number of nymphs and adults were counted for 4 days. Detached stem assay was performed on PI 567301B and Williams 82. The experiment was repeated three times over 3 months, with five replicates per genotype.

### Aphid Settling Preference

Aphid choice or settling preference assay was performed as previously described by Diaz-Montano et al. (2006), with a few modifications. Circular pots (15.2 × 14.6 cm) were used as choice test arenas. The arena consisted of two positions, with seeds of Williams 82 and PI567301B planted 10 cm apart in each arena. When all the plants reached the V1 stage, 150 mixed-aged apterous aphids were placed on a filter paper strip (3 × 8 cm) in the center of each arena (Diaz-Montano et al., 2006). The pots were placed far enough apart on a greenhouse bench to prevent aphids from moving between pots. The aphids were allowed to colonize the plants freely by walking from the filter paper to the plants. Aphid counts on each plant in each arena were recorded after 24 h. The experiment was conducted as a completely randomized block design with seven replications.

### Electrical Penetration Graph Analysis

The feeding behavior of the aphids on both whole plants and detached leaves of PI 567301B, and Williams 82 plants were determined by EPG analysis on a GIGA 8 complete system (EPG Systems, Wageningen, The Netherlands) as per Nalam et al. (2018). Adult apterous soybean aphids were starved 1 h before wiring and were wired on the dorsum with a 0.2-μm gold wire with the aid of water-based silver glue. The length of the gold wire was adjusted, such that it allowed the aphids to have free movement on the soybean leaf, and feeding was monitored for 8 h. For whole plants, an electrode (“plant electrode”) was inserted into the soil (Supplementary Figure 1A). For detached leaves, the electrode was placed into the microfuge tube containing the petiole immersed in water (Supplementary Figure 1B). For both the whole plant and detached leaf-feeding experiments, soybean plants at the V1 stage were used. The GIGA 8 system has eight channels that allow for the simultaneous recording of eight aphids feeding on eight plants. In the experimental setup, four channels recorded feeding behavior on PI 567301B plants, and four channels recorded feeding behavior on the susceptible Williams 82 plants. The entire EPG system and the experimental setup were placed in a Faraday cage to prevent the influence of external electromagnetic fields. Plants, detached leaves, and aphids were discarded after each experiment. Stylet+, the EPG acquisition software (EPG Systems, Wageningen, The Netherlands), was used to record waveforms for aphids feeding on whole plants or detached leaves and determine the amount of time spent on various feeding behaviors. The waveforms were categorized into five main phases: pathway or probing phase (C), non-probing phase (NP), sieve element phase (SEP), xylem phase (G), and derailed stylet phase (F), i.e., stylets having lost their proper position in the stylet bundle and therefore unable to penetrate normally (Tjallingii, 1988). The SEP can be further subdivided into the phloem salivation (E1) and phloem ingestion (E2) phases. Although E1 can occur by itself, the E2 phase is always preceded by the E1 phase. An Excel workbook developed by Sarria et al. (2009) was used to automatically calculate parameters that characterize soybean aphid feeding and probing behavior on the susceptible Williams 82 and resistant PI 567301B plants. There were 19 replicates each for Williams 82 and PI 567301B in the whole plant assays, and 20 replicates for Williams 82 and 23 replicates for PI 567301B in the detached leaf assays. Data were collected for all the treatments over 4 months. Recordings of aphids that did not show any feeding events and recordings in which aphids spent more than 70% of the recording time in the sum of NP, F, and G were discarded and not included in the analysis. The time spent in NP, C, SEP, and F, and the number of transitional events for each waveform were used to generate a behavioral kinetogram as described in Ebert et al. (2018).

### Reciprocal Grafting

Reciprocal grafting followed by a performance assay was performed to determine the source (root vs. shoot) of *Rag5*-mediated resistance (Joshi, 2017). Grafting was performed on 8-day-old soybean seedlings. A wedge-shaped cut was performed on the rootstock ~2–3 cm above the soil using a sterile razor. A corresponding V-shaped cut was performed on the scion 2–3 cm below the unifoliate leaves. The rootstock and the scion were aligned precisely and held together using grafting wax (Trowbridge's Grafting Wax, Eaton Bros. Corp., Hamburg, NY, United States) and clamped using a 1-cm long coffee straw cut longitudinally. The grafted plants were covered with plastic saran wrap to maintain high relative humidity and placed in the dark for 3 days, after which the grafted plants were moved to the greenhouse. The grafted plants were grown at 60% relative humidity, a temperature of 24–30°C, and a photoperiod of 14:10 (L:D) h. All the grafted plants were watered and fertilized, as mentioned previously. Grafts that successfully reached the fully opened first trifoliate stage were considered successful grafts. Grafted plants were grown for 4–5 weeks until the V1 stage before they were used to analyze aphid population growth or performance assay as described previously.

### Aphid Performance Assay on Detached Leaves Supplemented With kaempferol-9-Glycoside

Aphid performance, in response to kaempferol, was determined using a no-choice assay with detached leaves supplemented with kaempferol-9-glycoside (Sigma Aldrich, St. Louis, MO, United States). The setup was as mentioned previously for the detached leaf assay, except that kaempferol-9-glycoside was added to 1.5 ml of water in the microfuge tube for a final concentration of 10 mM. Kaempferol-9-glycoside was taken up systemically by the detached leaves. Three replications with PI567301B and Williams 82 were performed, with each experiment containing five replicates. Aphid population parameters, such the total numbers of adults and nymphs, were monitored and counted every day for 4 days, and data for day 4 are presented.

### Kaempferol Analysis by LC-MS/MS

To analyze kaempferol levels, leaf petiole exudates were collected from soybean, as described previously (Nachappa et al., 2016). Briefly, a single trifoliate leaf from soybean plants at the V2 stage was excised at the petiole base and weighed before exudate collection. Bacterial contamination was minimized by immersing the cut end immediately in 50% ethanol, followed by a 0.05% bleach solution. The cut trifoliate was then placed in 1 mM EDTA solution (pH 8) until three single trifoliate leaves were processed similarly. An additional 1 cm of the petiole was excised before transfer into a fresh solution of 1 mM (EDTA (4 ml) contained in a single well of a six-well tissue culture plate (Corning, Corning, NY). A total of three trifoliates were placed in each well. The entire setup was placed under 100% relative humidity for 24 h. Leaf petiole exudates from three wells were pooled and filtered through 0.2-μm pore size syringe filters (Millipore Sigma, Burlington, MA, United States) and lyophilized. A similar procedure was used to collect stem exudates, with one significant difference: stem exudates from V2 soybean plants were collected by excising at the base of the stem rather than at the petiole. Vascular sap-enriched leaf petiole and stem exudates were collected from control and soybean aphid-infested plants. Aphid infestation was performed by placing 10 adult aphids on each trifoliate for 24 h before exudate collection.

The lyophilized samples were reconstituted in 750 μl of 80% acetonitrile. The LC-MS/MS system consists of a Nexera X2 UPLC with 2 LC-30AD pumps, A SIL-30AC MP autosampler, a DGU-20A5 Prominence degasser, a CTO-30A column oven, and SPD-M30A diode array detector coupled to an 8040 quadrupole mass-spectrometer with ESI. For kaempferol detection, the MS was in negative mode [M-H]^−^ with an MRM optimized for: (a) 285.1 > 229 set for 100 ms dwell time with a Q1 pre-bias of 30 V, collision energy of 25 V, and Q3 pre-bias of 23 V; (b) 285.1 > 131.1 set for 100 ms dwell time with a Q1 pre-bias of 29 V, collision energy of 34 V, and Q3 pre-bias of 22 V, and (c) 285.1 > 239.05 set for 100 ms dwell time with a Q1 pre-bias of 12 V, collision energy of 27 V, and Q3 pre-bias of 15 V. The samples were chromatographed on a 100 × 4.6 mm Phenomenex Kinetex 2.6-μm Polar C18 100 Å (00D-4759-E0) maintained at 40°C. Solvent A consisted of water with 0.1% formic acid, and solvent B was acetonitrile with 0.1% formic acid. The solvent gradient was: 0–50% B, 6 −00% B, 9–100% B, 9.5–70% B, and 12–70% B. The flow rate was set at 0.4 ml/min, and the samples were analyzed as 1-μl injection volumes.

### RNA Isolation, cDNA Synthesis, and Quantitative Real-Time Reverse-Transcribed PCR

To determine if kaempferol biosynthesis is induced in response to aphid feeding, the expression of two genes putatively involved in flavonoid and kaempferol biosynthesis was analyzed in a time course assay spanning 24 h, during which the plants were sampled every 6 h. Gene expression was determined in both leaf and stem tissues collected from soybean plants at the V2 stage. Ten adult soybean aphids from the colony were placed on each trifoliate on the top of leaves of Williams 82 and PI 567301B. For leaf samples, the central trifoliate leaf on the youngest leaf was sampled at time 0, i.e., without aphid infestation, and at 6, 12, and 24 h post infestation (hpi). From the same plants, a 2-cm portion of the stem just below the youngest trifoliate was collected at 0, 6, 12, and 24 hpi. There were four plants per treatment. After collecting leaf and stem tissue, the plants were discarded. All aphids and nymphs were removed from the leaf and stem tissue before sample collection using a camel hair paintbrush. The 0 h time point samples were treated similarly. Leaf and stem tissue were immediately frozen in liquid nitrogen and later at −80°C until further processing.

Total RNA from the leaf and stem tissues was extracted using the Direct-Zol RNA Miniprep Kit (ZymoResearch, Irvine, CA, United States) following the protocol of the manufacturer, which included DNase treatment to eliminate DNA contamination. The samples were then quantified using a Nanodrop 1000 (Thermo Scientific, Wilmington, DE, United States). One microgram of RNA was used as a cDNA synthesis template using the First Strand cDNA synthesis kit (Gold Biotechnologies, St. Louis, MO, United States). cDNA synthesis was performed according to manufacturer protocol. For quantitative real-time reverse transcribed PCR (RT-qPCR), the cDNA was diluted at 1:50, and 5 μl was used in the reaction mixture. The total reaction volume was 25 μl and consisted of 12.5 μl SsoFastEvaGreen Supermix (Bio-Rad, Hercules, CA, United States), 0.125 μl forward/reverse primer (50 μM each), and 7.475 μl of molecular biology grade water. The cycling conditions used an initial denaturing step at 95°C for 5 min, followed by 40 cycles of 95°C for 10 s, 55°C for 30 s, followed by a melt curve analysis. Primer sequences, locus information, and amplicon lengths of the products are provided in Table 1. The PCR efficiencies of the target and internal control genes were determined using the LinRegPCR software (Ruijter et al., 2009) (Table 1). Reactions for all the samples were performed in triplicate, and the samples from four biological replicates were analyzed. Appropriate negative and positive controls were included in each run. The comparative C_T_ method (Schmittgen and Livak, 2008) was used to determine fold change. The C_T_ values of the genes of interest for each sample were first normalized to the internal control gene (*ELF-1B*), followed by normalization to the expression of the respective gene in Williams 82 0 h sample using the formula, 2^−Δ*ΔCT*^ (Schmittgen and Livak, 2008). Fold changes were log_2_ transformed to normalize data, and the log_2_ (fold change) data are presented and used for all statistical analyses.

**Table 1:** Quantitative real-time reverse transcribed PCR (RT-qPCR) primer pair sequences and corresponding PCR efficiencies.

**Gene**	**Functions**	**Glyma ID**	**Primer sequences (5' → 3')**	**PCR efficiency**	**Amplicon length (bp)**
**Internal control**					
*ELF-1B*	Eukaryotic elongation factor 1 beta	Glyma.02g44460	F: ACTCTGCACTCACCACTGCC R: AGGAAAGCTTGGAGCAAGTTGAG	2.00	247
**Kaempferol biosynthesis**					
*CHS7*	Chalcone synthase 7	Glyma.01g228870	F: TGAATGGGGTGTGTTGTTCG R: TGTTGTTGTTACAAACCCCAAGC	1.99	103
*FLS1*	Flavonol synthase 1	Glyma.13g082300	F: AAGCCTGCTGGGTCTGATTC R: AGGAAGGAGGCCACACAATG	2.04	112
**Rag5-candidate genes**					
*190200*	Protease family s26 mitochondrial inner membrane protease-related	Glyma.13g190200	F: TTCCGTTTTCCTCAGCAGGT R: CATCTGCTGCAAAACCCTTGC	1.95	152
*190500*	Protease family s26 mitochondrial inner membrane protease-related	Glyma.13g190500	F: GGTCTGCAGCAGCACTAGAA R: ATCCTGCAGAGGAAAACGGCA	1.97	212
*190600*	Unknown function	Glyma.13g190600	F: AACATGGAGGTGCCGTGATT R: CTTGCAACAAACCTCTCCGC	1.90	136

### Statistical Analysis

For all aphid population assays, means and standard errors were calculated for each response variable for each aphid-plant pair. The Anderson–Darling goodness-of-fit statistic (*P* ≤ 0.05) was used to determine if all data sets conform to the normality assumption of ANOVA. Datasets that did not conform to the assumptions of ANOVA, i.e., the whole plant and detached leaf assays and reciprocal graft performance assay, were rank transformed. For the three assays, a pairwise comparison between variables was made by Tukey's test. Data for the choice assay were analyzed as an ANOVA with a binary response count (i.e., aphids on a single plant were divided by total aphids on plants in the pot). Differences in transitional probabilities (used to construct the behavioral kinetogram) between the genotypes for each transitional event were analyzed by one-way ANOVA. For the analysis of EPG variables and the various aphid feeding behaviors, data were rank transformed since the data were not normally distributed, and differences between groups were determined by a one-way ANOVA. Data for the detached stem + leaves assays showed a normal distribution, and data were analyzed by a two-sample *t*-test without transformation. The log2-fold change for the RT-qPCR gene expression analysis of kaempferol biosynthesis and *Rag5* candidate genes between the two genotypes and at different time points was analyzed by a two-sample *t*-test at each time point. All data were analyzed using Minitab® 19 (Minitab, State College, PA, United States), other than the calculations of mean and standard error that were done using Microsoft Excel (Microsoft, Redmond, WA, United States).

## Results

### Rag5-Mediated Resistance Causes Reduced Aphid Populations on Whole Plants but Not on Detached Leaves

**Figure 1:**
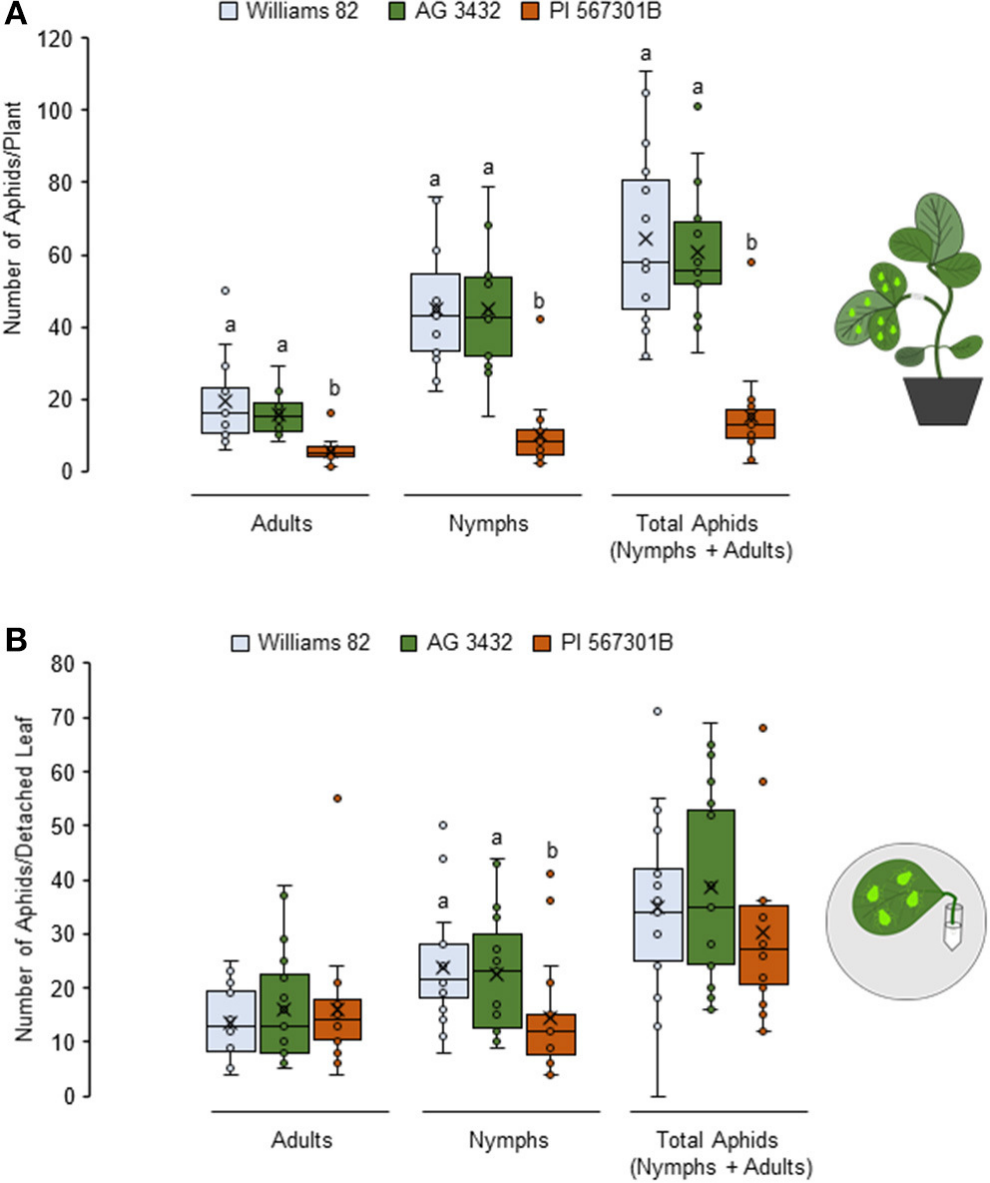
*Rag5*-mediated resistance to soybean aphids is lost in detached leaves. **(A)** Population of soybean aphids (total number of nymphs and apterous adults) on whole plants and **(B)** detached leaves of Williams 82, AG34332, and PI 567301B. The numbers of adults and nymphs were recorded daily for 4 days, and data for day 4 are shown. Three replications are performed for each genotype, and the experiment is repeated three times. Different letters above the bars indicate values that are significantly different. The cartoons represent the setup of the whole plant and detached leaf assays. Aphid size and number in the cartoons are not drawn to scale.

Resistance to soybean aphids has been observed in *Rag5-*carrying PI 567301B whole plants but not detached leaves (Michel et al., 2010). We confirmed this finding by no-choice bioassays on whole plants and detached leaves. In whole plant assays, number of adults (*P* < 0.05*, F*_2, 42_ = 14.16), nymphs (*P*< *0.0*5, *F*_2, 42_ = 25.62), and total aphid (*P* = 0.003, *F*_2, 42_ = 27.85) populations were lower on *Rag5* carrying plants as compared with the susceptible controls (Williams 82 and AG3432) (Figure 1A). On detached leaves, *Rag5* resistance did not influence the number of adults (*P* = *0.1*95, *F*_2, 42_ = 1.69) or the total number of aphids (*P* = 0.813, *F*_2, 42_ = 0.21) (Figure 1B); however, a lower number of nymphs (*P* = *0.0*05, *F*_2, 42_ = 5.84) were observed on plants carrying the *Rag5* gene.

### Rag5-Mediated Resistance Does Not Influence Aphid Settling Preference or Feeding Behavior

We evaluated soybean aphid settling preference for Williams 82 or *Rag5* carrying PI 567301B in whole-plant assays. Aphids did not show a preference for either genotype, and the proportion of aphids observed on Williams 82 (50.2 ± 3.4%, Mean ± SEM) and *Rag5* (49.7 ± 3.4%, Mean ± SEM) carrying plants was not different at 24 h post-release (*P* = 0.911, *F*_1, 12_ = 0.01).

**Figure 2:**
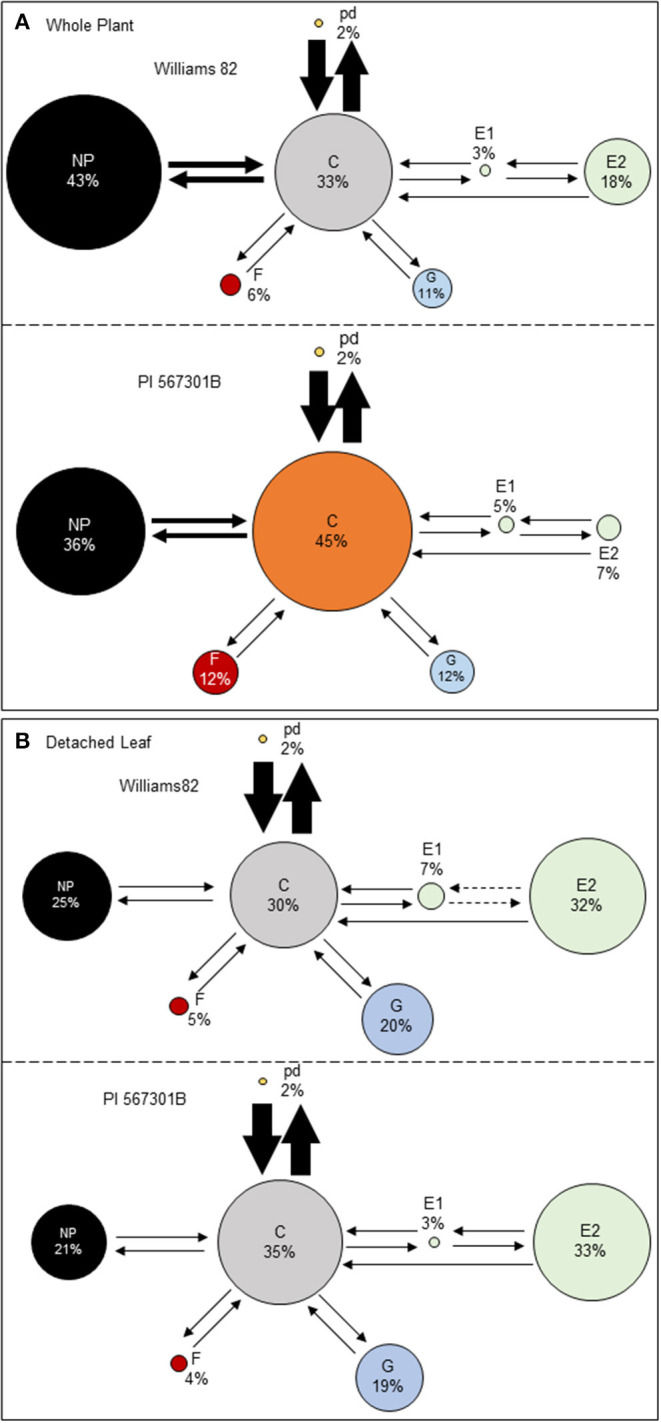
Aphid feeding behaviors on **(A)** whole plants and **(B)** detached leaves of Williams 82 and PI 563701B. The behavioral kinetogram shows aphid feeding behavior with arrows representing transitions and arrow thickness proportional to frequency. The size of the circles represents the percentage of time aphids spent in each of the waveforms: non-probing (NP), probing/pathway phase (C), potential drops (pd), derailed stylet (F), xylem phase (G), phloem salivation (E1), and phloem ingestion (E2). The data summarized are the summed counts from all aphids in each treatment. The durations or circle areas with different colors and dotted arrows represent parameters that are significantly different.

The EPG technique was used to determine if *Rag5-*mediated resistance influences aphid feeding. Soybean aphid feeding behavior was monitored on whole plants and detached leaves of Williams 82 and *Rag5* carrying PI 567301B. A behavioral kinetogram was constructed, which indicates the possible transitions to and from each waveform (Supplementary Table 1) and provides an overview of aphid feeding behavior (Figure 2). During aphid feeding, the non-probing (NP) phase always transitions into the probing/pathway phase (C). From probing, the aphid can transition back to non-probing, intracellular punctures, or potential drops (pd), xylem ingestion (G), derailed stylets (F), or phloem salivation (E1). The most common transition from probing on whole plants and detached leaves is to pd (Figures 2A,B). From pd, derailed stylets, and xylem ingestion, transitions back to probing can occur. From phloem ingestion, the aphid can transition to phloem salivation (E2) or back to probing. Finally, from phloem ingestion, the aphid can transition back to phloem salivation or return to probing. On whole plants, we did not observe any significant differences in the transitions from one phase to another between Williams 82 and *Rag5* carrying plants (Supplementary Table 1). On detached leaves, we observed fewer transitions from E1 to E2 and E2 to E1 on *Rag5* carrying plants compared with Williams 82 (Supplementary Table 1).

Aphid feeding behavior on whole plants of Williams 82 and *Rag5* carrying PI 567301B differed only in one parameter measured. A small but significant increase (1.3-fold) in the total time spent in probing on *Rag5* carrying plants compared with Williams 82 (Figure 2A, Table 2) was observed. There were no differences in aphid feeding in any of the major feeding behaviors (C, NP, SEP, and G) (Table 3) on detached leaves. Several parameters were evaluated to determine plant acceptability in the epidermal, mesophyll, and phloem tissues. Significant differences were observed for only two of the parameters evaluated on detached leaves. The number of E2 waveforms was 1.9-n-fold lower on *Rag5* carrying plants than Williams 82 (Table 2). However, no differences were observed in the total time spent in E2 (Table 2). Two parameters, the potential phloem ingestion index (potential E2 index) and the percent time spent in sustained E2 (i.e., E2 > 10 min), have been used previously to characterize phloem-based resistance to aphids (Girma et al., 1992). Both parameters provide a measure of the time spent in sustained E2 after the aphid initiates the first E1 phase. However, no differences were observed for both parameters on both whole plants and detached leaves (Tables 2 and 3). The second parameter showing a difference between the two genotypes on detached leaves was the mean duration of non-probing, which was 2.4-fold higher in Williams 82 compared with *Rag5* carrying plants (Supplementary Table 2). The findings suggest that the *Rag5*-mediated resistance in PI 567301B did not influence aphid settling preference and did not affect aphid feeding behavior.

**Table 2:** Feeding behaviors of aphids on whole plants of Williams 82 and PI 567301B.

**Parameters**		**Williams 82^a^**	**PI 567301B^a^**	***P*-value^b^**	***F*-value^c^**
		***N* = 19**	***N* = 19**		
**Probing behavior**					
Time Spent in non-probing	(min)	205.7 ± 25.4	170.5 ± 19.2	0.293	*F*_1, 36_ = 1.14
Time to 1^st^ Probe	(min)	18.9 ± 8.8	15.8 ± 7.2	0.796	*F*_1, 36_ = 0.07
Number of probes		33.5 ± 6.5	33.0 ± 4.3	0.556	*F*_1, 36_ = 0.35
Total probing time	(min)	274.3 ± 25.4	309.5 ± 19.2	0.293	*F*_1, 36_ = 1.14
Total duration of C	(min)	157.3 ± 17.5	214.8 ± 16.7	***0.0015***	*F*_1, 36_ = 6.5
Number of potential drops (pd)		102.8 ± 15.8	110.6 ± 11.0	0.566	*F*_1, 36_ = 0.33
Total duration of pd	(min)	9.0 ± 1.2	10.8 ± 1.1	0.365	*F*_1, 36_ = 0.84
**Xylem feeding**					
Aphids with xylem phase	(%)^d^	89 (17/19)	68 (13/19)	n.s.	
Number of G		1.9 ± 0.3	3.8 ± 1.1	0.872	*F*_1, 28_ = 0.03
Time spent in G	(min)	51.3 ± 20.2	57.1 ± 13.7	0.140	*F*_1, 28_ = 2.31
**Sieve element phase**					
Aphids with SEP	(%)^d^	74 (14/19)	79 (15/19)	n.s.	
Time in SEP	(min)	83.0 ± 35.5	46.6 ± 8.6	0.551	*F*_1, 27_ = 0.36
Number of E1 waveforms		2.7 ± 0.6	3.3 ± 0.8	0.760	*F*_1, 27_ = 0.09
Time to 1^st^ E1	(min)	243.1 ± 39.1	216.2 ± 42.5	0.378	*F*_1, 27_ = 0.80
Total duration of E1	(min)	13.7 ± 3.3	21.5 ± 4.8	0.264	*F*_1, 27_ = 1.3
Number of E2 waveforms		1.5 ± 0.6	1.4 ± 0.4	0.775	*F*_1, 27_ = 0.08
Total duration of E2	(min)	88.2 ± 44.7	31.4 ± 8.3	0.523	*F*_1, 27_ = 0.42
Time to 1^st^ E2	(min)	337.5 ± 36.6	356.4 ± 31.1	0.931	*F*_1, 27_ = 0.01
Potential E2 Index	(%)	27.5 ± 11.0	22.7 ± 8.9	0.882	*F*_1, 21_ = 0.02
Percent time in sustained E2	(%)	44.8 ± 14.7	34.7 ± 12.7	0.701	*F*_1, 21_ = 0.15

*One-way ANOVA was performed to analyze the effects of treatments on each parameter (after rank transformation of the raw data)*.

a *Data presented are the means ± standard error of mean for aphids that displayed the behaviors*.

b *P-values that are significant are highlighted in italics and are also bolded*.

c *The degrees of freedom for xylem feeding and sieve element phase vary from the total number of samples, since not all aphids displayed xylem and sieve element feeding*.

d *The z-test was performed to compare proportions, and n.s. indicates no significant differences*.

**Table 3:** Feeding behaviors of aphids on detached leaves plants of Williams 82 and PI 567301B.

**Parameters**		**Williams 82^a^**	**PI 567301B^a^**	***P*-value^6^**	***F*-value^c^**
		***N* = 20**	***N* = 23**		
**Probing behavior**					
Time Spent in non-probing	(min)	117.9 ± 18.9	100.7 ± 19.3	0.414	*F*_1, 41_ = 0.74
Time to 1^st^ Probe	(min)	26.4 ± 13.1	6.1 ± 1.9	0.227	*F*_1, 41_ = 0.07
Number of probes		16.2 ± 2.5	21.3 ± 3.8	0.549	*F*_1, 41_ = 0.37
Total probing time	(min)	362.1 ± 18.9	378.8 ± 19.4	0.414	*F*_1, 41_ = 0.68
Total duration of C	(min)	143.0 ± 17.8	167.1 ± 16.5	0.237	*F*_1, 41_ = 1.44
Number of potential drops (pd)		100.2 ± 13.2	113.0 ± 12.2	0.394	*F*_1, 41_ = 0.74
Total duration of pd	(min)	575.2 ± 71.0	653.3 ± 71.0	0.487	*F*_1, 41_ = 0.49
**Xylem feeding**					
Aphids with xylem phase	(%)^d^	70 (14/20)	74 (17/23)	n.s.	
Number of G		2.1 ± 0.4	2.3 ± 0.5	0.930	*F*_1, 29_ = 0.01
Time spent in G	(min)	94.5 ± 18.2	89.8 ± 17.4	0.871	*F*_1, 29_ = 0.03
**Sieve element phase**					
Aphids with SEP	(%)^d^	90 (18/20)	96 (22/23)	n.s.	
Time in SEP	(min)	163.4 ± 30.5	152.0 ± 27.5	0.709	*F*_1, 38_ = 0.14
Number of E1 waveforms		4.9 ± 0.6	3.1 ± 0.5	0.070	*F*_1, 38_ = 3.45
Time to 1^st^ E1	(min)	195.4 ± 29.5	144.8 ± 23.0	0.209	*F*_1, 38_ = 1.63
Total duration of E1	(min)	35.4 ± 15.2	12.2 ± 2.3	0.177	*F*_1, 38_ = 1.89
Number of E2 waveforms		3.9 ± 0.5	2.1 ± 0.2	***0.002***	*F*_1, 38_ = 4.88
Total duration of E2	(min)	135.5 ± 25.3	146.5 ± 27.6	0.989	*F*_1, 38_ = 0.00
Time to 1^st^ E2	(min)	248.3 ± 30.5	201.1 ± 27.7	0.160	*F*_1, 38_ = 2.05
Potential E2 Index	(%)	50.9 ± 8.6	49.4 ± 8.4	0.908	*F*_1, 36_ = 0.01
Percent time in sustained E2	(%)	56.5 ± 8.5	65.5 ± 8.3	0.357	*F*_1, 36_ = 0.87

*One-way ANOVA was performed to analyze the effects of treatments on each parameter (after rank transformation of the raw data)*.

a *Data presented are means ± standard error of mean for aphids that displayed the behaviors*.

b *P-values that are significant are highlighted in italics and bold*.

c *The degrees of freedom for xylem feeding and sieve element phase vary from the total number of samples, since not all aphids displayed xylem and sieve element feeding*.

d *The z-test was performed to compare proportions, and n.s. indicates no significant differences*.

### Stem as a Potential Source of Rag5-Mediated Resistance

**Figure 3:**
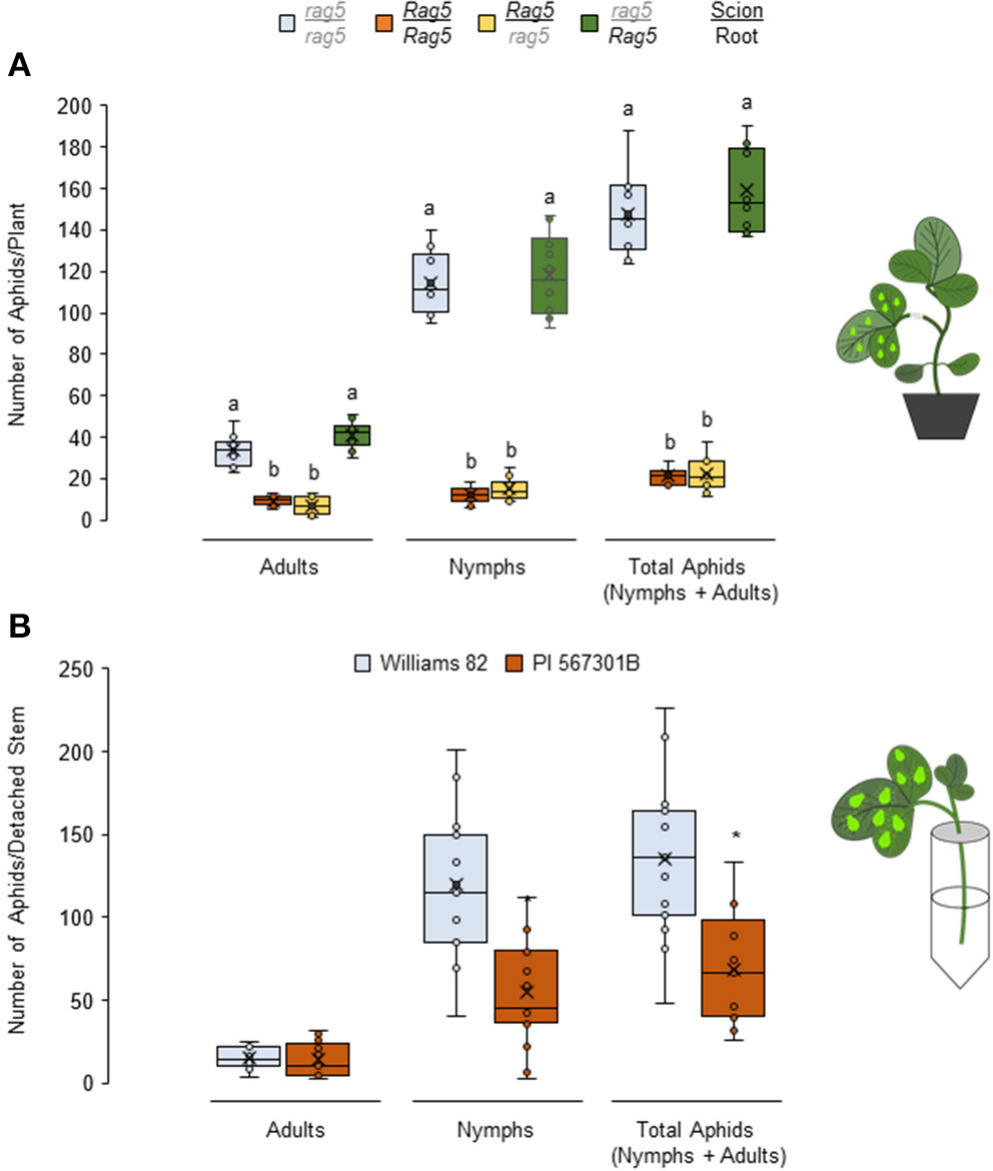
*Rag5-*mediated resistance to soybean aphids is derived from the stems. Populations of soybean aphids in **(A)** chimeric reciprocally grafted plants and in **(B)** detached stems of Williams and PI 567301B. For both **(A,B)**, the numbers of adults and nymphs are recorded daily for 4 days, and data for day 4 are shown. In **(A)**, the values are mean ± standard error of mean from 10 plants that showed successful graft formation. Different letters above the bars indicate values that are significantly different from each other (*P* < 0.05; GLM/ANOVA). In **(B)**, three replications are performed for each genotype, and the experiment is repeated three times. Asterisks indicates values that are significantly different from each other (*P* < 0.05; *t*-test). The cartoons represent the setup of the reciprocal graft and detached stem assays. Aphid size and number in the cartoons are not drawn to scale.

To determine the source of *Rag5*-mediated resistance, reciprocal grafting experiments were performed to generate chimeric *Rag5/rag5* plants that contained PI 567301B scions (shoot) and Williams 82 rootstock, and *rag5/Rag5* plants that contained Williams 82 scions and PI 567301B rootstock. *Rag5*/*Rag5* (PI 567301B as scion and rootstock) and *rag5*/*rag5* (Williams 82 as scion and rootstock) self-grafted plants were used as resistant and susceptible controls, respectively. In no-choice assays using the self-grafted plants, total number of aphids (*P* < 0.05, *F*_3, 36_ = 47.65) including the number of adults (*P* < 0.05, *F*_3, 36_ = 43.88) and nymphs (*P* < 0.05, *F*_3, 36_ = 41.88) (Figure 3A) were lower on *Rag5*/*Rag5* (Figure 3A) plants as compared with *rag5*/*rag5* plants. These results show the same pattern as in ungrafted plants (Figure 1A). We expected that if the roots were the source of *Rag5*-mediated resistance, aphid populations would be lower on graft combinations where the *Rag5* gene is present in rootstocks. Contrary to the expectations, aphid populations were lower in the *Rag5*/*rag5* chimera (PI 567301B scion/Williams 82 rootstock, Figure 3A). By contrast, the aphid population in the *rag5*/*Rag5* chimera (Williams 82 scion/PI 567301B rootstock) was comparable with the aphid populations observed on the *rag5*/*rag5* plant and higher than on *Rag5*/*Rag5* plants. These results suggest that *Rag5-*mediated resistance in PI 567301B is derived from the shoots and not the roots.

Given that *Rag5-*mediated resistance is lost in detached leaves and the *Rag5*/*rag5*(PI 567301B scion/Williams 82 rootstock) chimera but present in the *rag5*/*Rag5* (Williams 82 scion/PI 567301B rootstock) chimera, we hypothesized that the resistance factor in *Rag5* carrying PI 567301B is derived from the stem. The hypothesis was verified by determining aphid performance on detached stem + leaves of Williams 82 and PI 567301B (Figure 3B). The total number of aphids is lower on *Rag5* carrying plants than the susceptible variety, Williams 82 (*P* < 0.05, *F*_1, 28_ = 16.71). The number of nymphs was also lower on *Rag5* containing detached stems (*P* < 0.05, *F*_1, 28_ = 19.03). However, the total number of adults did not differ (*P* = *0.7*88, *F*_1, 28_ = 0.09). Taken together, the reciprocal grafting and detached stem + leaves assays suggest that the possible site of *Rag5*-mediated resistance is the stem.

### Kaempferol Potentially Mediates *Rag5* Resistance

**Figure 4:**
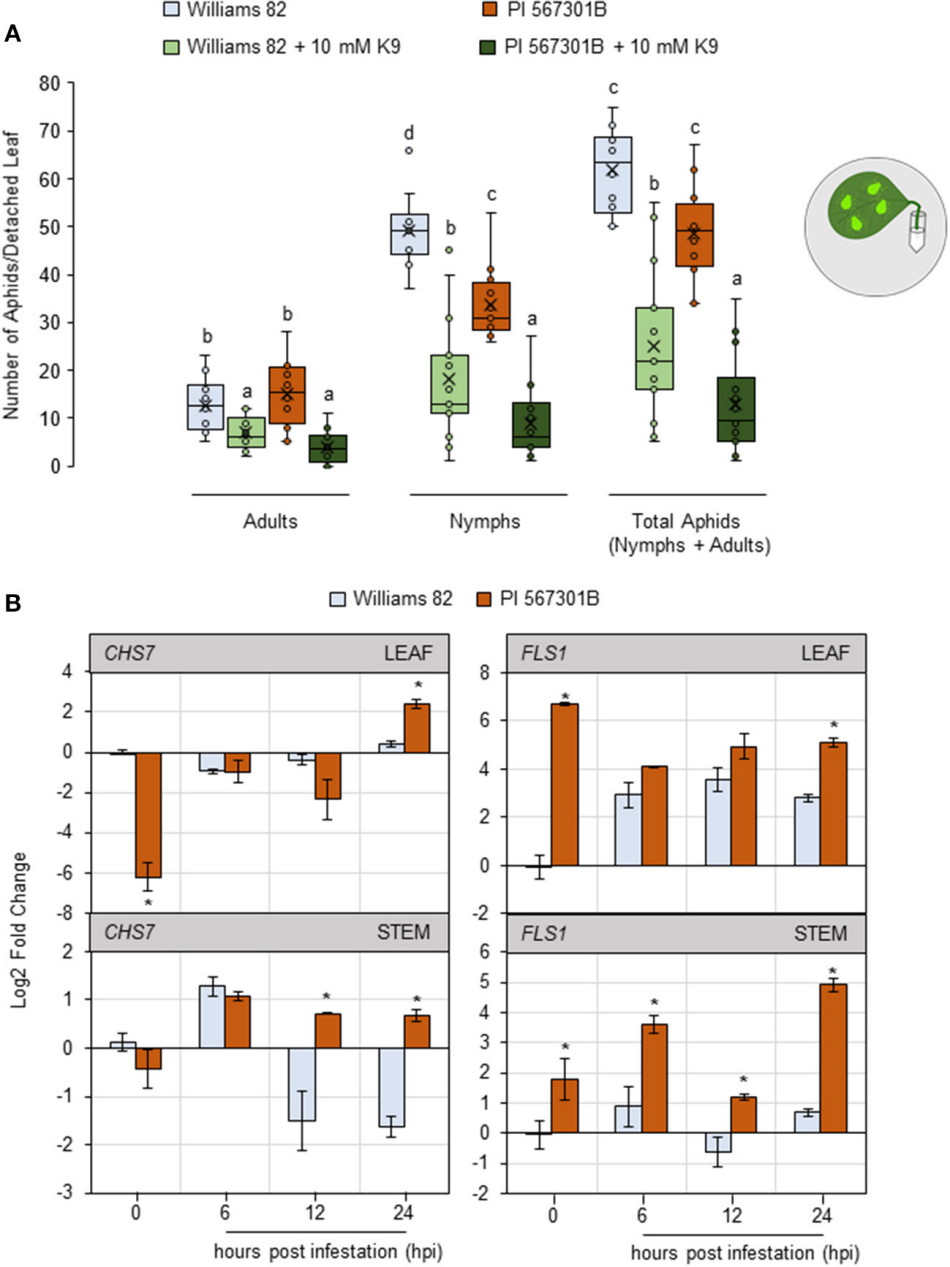
Kaempferol may play a role in *Rag5-*mediated resistance to soybean aphids. **(A)** Populations of soybean aphids on detached leaf assays supplemented with kaempferol-9-glycoside (K9). The numbers of adults and nymphs are recorded daily for 4 days, and data for day 4 are shown. The values are mean ± standard error of mean (*n* = 12). Different letters above the bars indicate values that are significantly different from each other (*P* < 0.05; GLM/ANOVA). Three replications are performed for each genotype and treatment combination, and the experiment is repeated three times. The cartoon represents the setup for the detached leaf assay. Aphid size and number in the cartoons are not drawn to scale. **(B)** Quantitative real-time reverse transcribed PCR (RT-qPCR) to determine the expression of kaempferol biosynthesis genes, chalcone synthase 7 (*CHS7*, Glyma01g228700), and flavanol synthase 1 (*FLS1*, Glyma13g082300) in leaf and stem tissue of Williams 82 and PI 567301B in a time course experiment. The comparative C_T_ method is used to calculate fold change, and all samples are compared with Williams 82 0 h (uninfested samples). Data are presented as log2-fold change, and the error bars represent standard deviation (*n* = 4). Asterisks above the bars indicate statistically significant differences (*P* < 0.05; *t*-test).

Isoflavones are important for aphid defense in *Rag*-containing plants (Hohenstein et al., 2019). The isoflavone kaempferol-9- glycoside is induced in *Rag5-*containing plants (Mian, 2014). A detached leaf assay was performed by supplementing leaves with 10 mM of kaempferol-9-glycoside to determine if the isoflavone impacts aphids. Supplementing with kaempferol-9-glycoside reduced the numbers of adults (*P* < 0.05, *F*_1, 47_ = 36.28), nymphs (*P* < 0.05, *F*_1, 47_ = 93.89), and total aphids (*P* < 0.05, *F*_1, 47_ = 93.93), irrespective of the genotype (Figure 4A).

The expression of two genes involved in kaempferol biosynthesis, chalcone synthase 7 (*CHS7*, Glyma01g228700) and flavonol synthase 1 (*FLS1*, Glyma13g082300) (Nagamatsu et al., 2007; Nakata et al., 2016) was monitored in leaf and stem tissue over a 24-h period. *CHS7* is involved in converting *p*-coumaryl CoA to naringin chalcone, one of the first steps in kaempferol biosynthesis (Saito et al., 2013). In uninfested leaf samples, the expression of *CHS7* was 6-fold lower in *Rag5* carrying plants compared with Williams 82 (Figure 4B). At 24 h post infestation (hpi), the expression of *CHS7* was higher in *Rag5* carrying plants than in Williams 82 (Figure 4B). In stem tissue, the expression of *CHS7* was induced in both genotypes within 6 hpi and remained upregulated at 12 and 24 hpi only in *Rag5* carrying plants (Figure 4B). *FLS1* is involved in the conversion of dihydrokaempferol to kaempferol (Saito et al., 2013). The expression of *FLS1* was higher in un-infested leaves of *Rag5* carrying plants compared with Williams 82. In response to aphid infestation, the expression of *FLS1*was higher only at 24 hpi (Figure 4B). On the other hand, in the stems, *FLS1* was upregulated at all time points (Figure 4B).

As aphids feed on plant phloem and xylem, the presence of kaempferol was monitored in vascular sap-enriched petiole and stem and xylem exudates. Exudates collected from un-infested and aphid-infested plants were evaluated for the presence of kaempferol by GC-LC/MS. However, no kaempferol was detected in any of the exudate fractions (data not shown). The data of the authors show that the resistance lost in *Rag5* carrying detached leaves is restored by supplementing 10 mM kaempferol, suggesting that isoflavanol has an antibiotic influence on aphids. Coupled with the observation that kaempferol biosynthesis is upregulated in both leaves and stems during aphid infestation, it is plausible that *Rag5*-mediated resistance involves kaempferol.

### Glyma13g190600 Is a Potential *Rag5* Candidate

**Figure 5:**
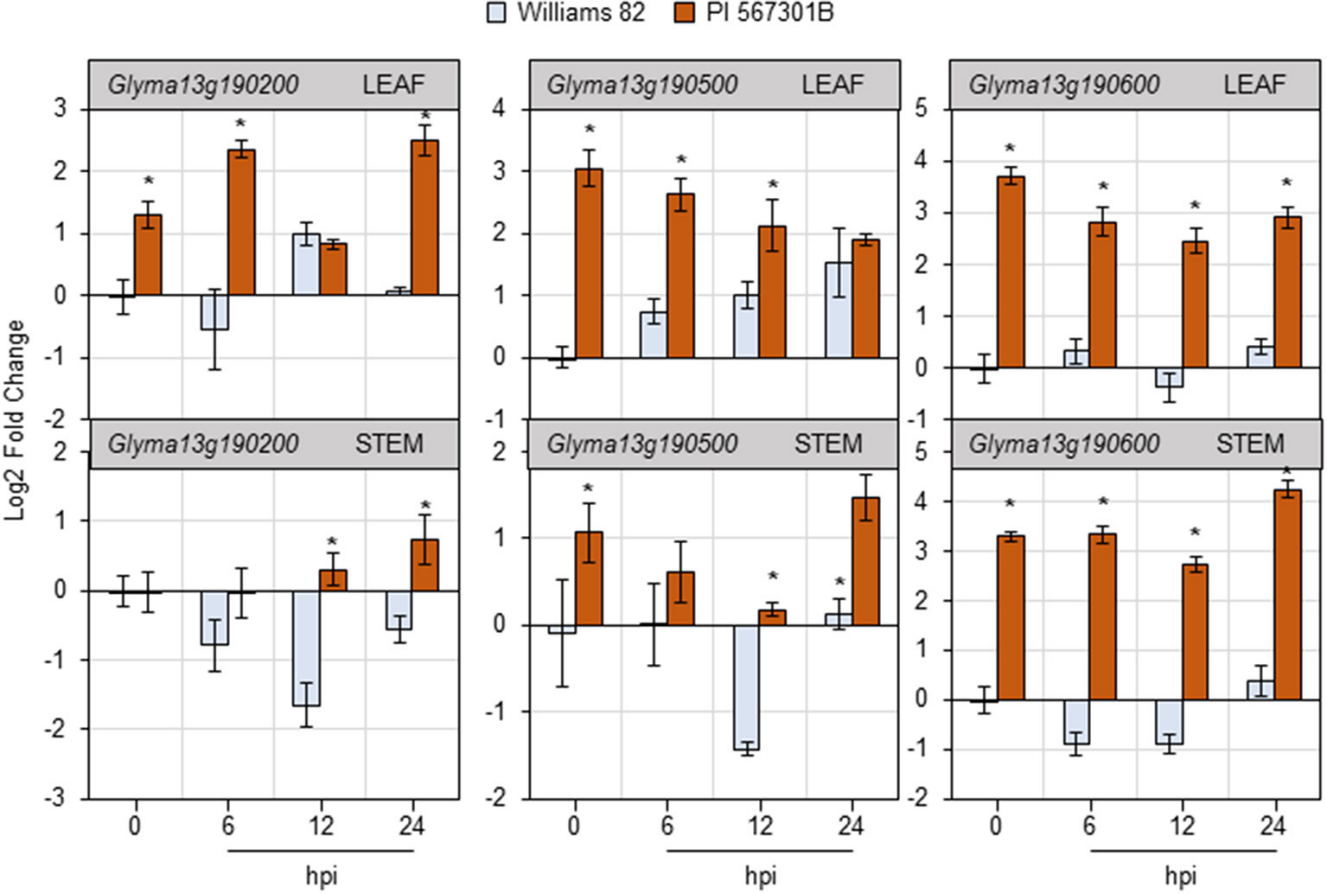
Soybean feeding induces expression of putative *Rag5* candidate genes in leaves and stems. Quantitative real-time reverse transcribed PCR (RT-qPCR) to determine the expression of three putative *Rag5* candidate genes, Glyma13g190200, Glyma13g190500, and Glyma13g190600, in leaf and stem tissue of Williams 82 and PI 567301B in a time course experiment. The comparative C_T_ method is used to calculate fold change, and all samples are compared with Williams 82 0 h (uninfested samples). Data are presented as log2-fold change, and the error bars represent standard deviation (*n* = 4). Asterisks above the bars indicate statistically significant differences (*P* < 0.05; *t*-test).

Three non-NBS-LRR genes present in the *Rag5-*containing QTL region on chromosome 13 have been implicated in *Rag5*-mediated resistance (Lee et al., 2017). The expression of the three genes—Glyma13g190200, Glyma13g190500, and Glyma13g190600—was monitored in the leaves and stems of Williams 82 and *Rag5* carrying PI 567301B in a time-course experiment in response to aphid feeding by RT-qPCR (Figure 5). In the leaves of soybean plants, the expression of all the three genes was higher in *Rag5* carrying plants without aphid infestation (0 hpi, hours post infestation). Two of the putative *Rag5*-candidates—Glyma13g190500 and Glyma13g190600—showed greater than a 2-fold higher expression at 0 hpi in *Rag5* carrying plants compared with Williams 82. For Glyma13g190200, higher expression was observed at 6 and 24 hpi in *Rag5* carrying plants. For Glyma13g190500, the expression gradually increased in Williams 82 over the 24-h period and reduced in *Rag5* carrying plants. On the other hand, low expression was observed for Glyma13g190600 in Williams 82, and a > 2-fold expression was observed in response to aphid infestation in *Rag5* carrying plants at 6, 12, and 24 hpi. In the stems, no significant differences in expression were observed for Glyma13g190200 (Figure 5). For Glyma13g190500, significant downregulation was observed at 12 hpi in Williams 82, and significant upregulation was observed at 24 hpi (Figure 5). Of the three genes, Glyma13g190600 showed significant upregulation at 0, 6, 12, and 24 hpi in *Rag5* plants.

## Discussion

Host plant resistance is an economical and sustainable strategy for managing soybean aphids (Hesler et al., 2013). However, a breakdown in resistance due to the emergence of virulent soybean aphid biotypes has been a major limiting factor for utilizing host plant resistance (Natukunda and MacIntosh, 2020). Hence, characterizing resistance mechanisms will help build a mechanistic understanding of soybean-soybean aphid interactions and inform strategies to identify and breed or engineer more durable resistance sources. To the knowledge of the authors, this is the first study to characterize the nature of *Rag5-*mediated resistance at several biological levels: ecological (aphid performance, settling preference, and feeding behavior); physiological (kaempferol content); and transcriptional (gene expression analysis).

Host plant resistance mechanism can be antibiosis (adverse impacts on insect biology), antixenosis (adverse impacts on insect behavior), and tolerance (similar yield in the presence or absence of insect pressure) (Painter, 1951; Kogan and Ortman, 1978; Smith, 2005; Natukunda and MacIntosh, 2020). No-choice tests or aphid performance growth assays have been performed to determine antibiosis, and choice tests have been performed to establish antixenosis (Diaz-Montano et al., 2006). The performance and preference assays suggest that *Rag5* carrying PI 567301B has an antibiosis mode of resistance. The EPG technique has been used to characterize differences in the feeding behavior of soybean aphids colonizing resistant and susceptible plants (Diaz-Montano et al., 2007; Crompton and Ode, 2010; Chandran et al., 2013; Todd et al., 2016). For instance, on *Rag1* carrying plants, a gene that confers antibiosis (Hill et al., 2006a; Mian et al., 2008), fewer aphids can reach the phloem, and those that do take a longer time to reach the first sieve element. These aphids also spend significantly shorter time feeding from the phloem, suggesting undetermined antibiotic factor(s) are present in the phloem of *Rag1* plants (Crompton and Ode, 2010). Interestingly, although fewer aphids can reach the phloem and took longer to reach the first sieve element, the time spent in phloem-feeding was not affected by *Rag2-*mediated resistance (Todd et al., 2016; Baldin et al., 2018). In this study, the only significant difference in feeding behavior on whole plants was that aphids exhibited a longer duration of probing activity on plants carrying the *Rag5* gene than on Williams 82. During probing, aphid stylets probe and sample epidermal and mesophyll cell content and longer probing suggest low plant acceptability and anti-xenosis type of resistance. Interestingly, PI 567301B was earlier identified to have a combination of antibiosis and antixenosis modes of resistance (Mian et al., 2008). Collectively, results from the aphid performance and preference assays and EPG analysis of feeding behaviors suggest both antibiosis and antixenosis modes of resistance in *Rag5* carrying PI 567301B.

Aphid populations were lower on *Rag5* plants than the susceptible control, while the antibiosis effect was absent when the experiment was conducted using detached leaves. The use of detached leaves has been proposed as a more rapid and practical assay to screen germplasm for resistance to aphids. Several studies have shown no significant differences in aphid performance on detached leaves than intact plants, but these studies used only susceptible plants (MacKinnon, 1961; Nam and Hardie, 2012; Soffan and Aldawood, 2014; Li and Akimoto, 2018). When detached leaf assays are performed with resistant plants, contrasting results are observed. For instance, soybean aphid performance on detached soybean leaves is genotype-dependent (Michel et al., 2010). Resistance observed in whole plants of *Rag2* plants is retained in detached leaves, but resistance in whole plants of *Rag5* plants is lost in detached leaves. A similar observation was also reported for greenbugs (*Schizaphis graminum*), which grew poorly on intact leaves of three resistant varieties of sorghum (*Sorghum bicolor*) but performed better on detached leaves of the same varieties (Montllor et al., 2002). To summarize, *Rag5*-mediated resistance is likely derived from a source other than the leaf.

In soybeans, attacks by pathogens and/or herbivores result in the accumulation of isoflavones in leaves. Examples of isoflavones that accumulate include daidzein, formononetin, genistein, glycitein, and glyceollins (Ingham et al., 1981; Osman and Fett, 1983; Wegulo et al., 2005; Lygin et al., 2013; Murakami et al., 2014; Hohenstein et al., 2019; Yao et al., 2020). During susceptible interactions, soybean aphid infestation leads to increased isoflavone biosynthesis and accumulation during both the short-term (Yao et al., 2020) and long-term colonization of plants (Hohenstein et al., 2019). It has been demonstrated that *Rag5* resistance is correlated with levels of the isoflavone, kaempferol (Mian, 2014). In this study, supplementing kaempferol-9-glycoside in detached leaf assays reduced aphid populations on detached leaves of *Rag5* carrying plants, but the resistance was absent from untreated detached leaves. Further, aphid feeding upregulated the expression of two genes involved in flavonoid and kaempferol biosynthesis consistently in stem tissues of *Rag5* plants compared with leaves. Intriguingly, the requirement of stems for *Rag5*-mediated resistance, as evidenced from the reciprocal grafting experiments and detached stem assays, suggests that stems are required for kaempferol biosynthesis in the leaves. During feeding on plants, aphids secrete watery saliva that contains salivary effectors in the form of mRNA transcripts, proteins, and metabolites that modulate host physiology to benefit the insect and facilitate sustained feeding (Chen et al., 2020). These aphid salivary effectors are present in the phloem and can be perceived by plants in tissues other than those being infested. For instance, green peach aphid feeding on leaf tissue can induce oxylipin biosynthesis in the roots (Nalam et al., 2012). We hypothesize that during soybean aphid feeding, salivary effectors secreted into leaf tissue move systemically and activate an as yet undetermined defense response in the stems.

It has been proposed that isoflavones are part of a non-phloem defense mechanism against soybean aphids, as they tend to accumulate in the parenchyma or epidermal cells in response to aphid feeding (Hohenstein et al., 2019). We did not detect kaempferol in phloem-sap enriched stem and petiole exudates, which suggests that kaempferol is not found in the vasculature. There were no differences in aphid feeding in SEP, but we observed an increase in probing on *Rag5* plants. The presence of isoflavones in parenchyma cells and the increased time spent in probing on *Rag5* plants suggest that the aphids encounter and ingest isoflavones during probing. Collectively, the findings indicate that *Rag5*-mediated resistance is derived from the shoots and involves kaempferol.

NBS-LRR genes play an important role in plant defense (DeYoung and Innes, 2006). By RNA-seq analysis of resistant and susceptible near-isogenic lines (NILs) developed for the *Rag5* locus, Lee et al. (2017) showed that LRR-type genes may not be responsible for *Rag5-*conferred aphid resistance in soybean leaves. We hypothesized that the LRR-type genes found in the *Rag5*-containing QTL would show differential expression in the stem tissue and not leaves. Of the 13 candidate genes in the *Rag5*-containing QTL, only three showed differential expression in response to aphid infestation in this study. Two of these genes have the annotated function “protease family S25 mitochondrial inner membrane protease.” The mitochondrial inner membrane proteases are required for the maturation of mitochondrial proteins delivered to the inner membrane space (Ghifari et al., 2019; Ruan et al., 2020). In *Arabidopsis, FtSH4*, a mitochondrial protease, regulates WRKY-dependent salicylic acid accumulation and signaling (Zhang et al., 2017), with increased levels of salicylic acid observed in *FtSH4* knockouts. Previously, it has been shown that jasmonate-dependent plant defenses mediate soybean response to aphid infestation (Studham and MacIntosh, 2013; Selig et al., 2016). It is plausible that an increase in the expression of both the genes results in a suppression of salicylic acid-mediated signaling, resulting in an increase in jasmonate-mediated responses and resistance to soybean aphids. Whether this occurs can be the focus of future research efforts.

Interestingly, the third gene—Glyma13g190600—encodes a peptide of 93 amino acids and is annotated as an unknown function. Stringent BLAST searches with the peptide sequence did not reveal a homolog in any eukaryotic species, nor were we able to identify any conserved domains in the protein. BLASTN with the coding sequence identified a predicted subtilisin-like protease (XM_017565204.1) with only 30% query coverage (at the 3' end of the sequence) and an E-value of 1^−20^. However, it is important to keep in mind that the genome sequence of soybean is only available for Williams 82, and that the genotype may not possess a functional allele of the gene. The gene showed the highest expression levels in both leaves and stems in plants carrying the *Rag5* gene, suggesting that Glyma13g190600 could plausibly represent a novel form of resistance to aphids and warranting further investigation.

This study demonstrated that *Rag5-*mediated resistance is derived from the stem and not the leaves; hence detached leaves alone should not be used for screening novel sources of resistance. We show that isoflavones, such as kaempferol, and potentially other chemical defenses are involved in *Rag5* resistance response. Future research may aim to correlate transcriptomic and metabolomic responses in stem vs. leaf tissues with aphid performance and behavior on *Rag5* and susceptible genotypes to understand mechanisms underlining *Rag5*-mediated resistance. A better understanding of potential mechanisms of *Rag* genes will inform strategies to confer broad and durable resistance to soybean aphids.

## Data Availability Statement

The raw data supporting the conclusions of this article will be made available by the authors, without undue reservation.

## Author Contributions

VN conceived and designed the experiments. KJ, JB, MM, SA, and EB performed the experiments. FD and VN performed the LC-MS/MS analysis. PN and VN analyzed the data and wrote the manuscript. VN contributed reagents, consumables, and the use of equipment in his laboratory. All authors contributed to the article and approved the submitted version.

## Conflict of Interest

The authors declare that the research was conducted in the absence of any commercial or financial relationships that could be construed as a potential conflict of interest.
