# Supplementary material for: The Source of Rag5-Mediated Resistance to Soybean Aphids Is Located in the Stem
Source: Front Plant Sci. 2021 Jul 16;12:689986. doi: 10.3389/fpls.2021.689986 (PMC8322969; doi:10.3389/fpls.2021.689986)
Supplement: Supplementary file 1 [file Data_Sheet_1.docx]

Supplementary Material

## Supplementary Figures


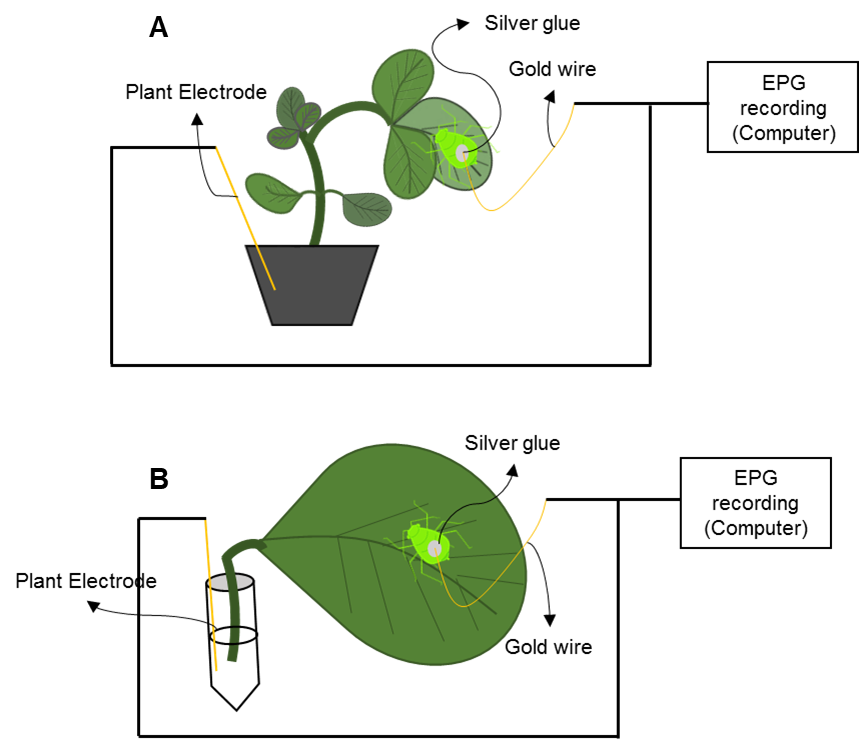


**Supplementary Figure 1.** Experimental set-up for Electrical Penetration Graph analysis of soybean aphid feeding behavior on (A) whole plants and (B) detached leaves.
